# Multi-Functional Regulation of 4E-BP Gene Expression by the Ccr4-Not Complex

**DOI:** 10.1371/journal.pone.0113902

**Published:** 2015-03-20

**Authors:** Hirokazu Okada, Ralf B. Schittenhelm, Anna Straessle, Ernst Hafen

**Affiliations:** 1 Institute of Molecular Systems Biology, Swiss Federal Institute of Technology (ETH) Zurich, Wolfgang Pauli Str. 16, 8093, Zürich, Switzerland; 2 Department of Biochemistry and Molecular Biology, School of Biomedical Sciences, Faculty of Medicine, Nursing and Health Sciences, Monash University, Clayton Campus, Wellington Road, Clayton, Victoria, 3800, Australia; Instituto Gulbenkian de Ciência, PORTUGAL

## Abstract

The mechanistic target of rapamycin (mTOR) signaling pathway is highly conserved from yeast to humans. It senses various environmental cues to regulate cellular growth and homeostasis. Deregulation of the pathway has been implicated in many pathological conditions including cancer. Phosphorylation cascades through the pathway have been extensively studied but not much is known about the regulation of gene expression of the pathway components. Here, we report that the mRNA level of eukaryotic translation initiation factor (eIF) subunit 4E-binding protein (4E-BP) gene, one of the key mTOR signaling components, is regulated by the highly conserved Ccr4-Not complex. RNAi knockdown of Not1, a putative scaffold protein of this protein complex, increases the mRNA level of 4E-BP in *Drosophila* Kc cells. Examination of the gene expression mechanism using reporter swap constructs reveals that Not1 depletion increases reporter mRNAs with the 3’UTR of 4E-BP gene, but decreases the ones with the 4E-BP promoter region, suggesting that Ccr4-Not complex regulates both degradation and transcription of 4E-BP mRNA. These results indicate that the Ccr4-Not complex controls expression of a single gene at multiple levels and adjusts the magnitude of the total effect. Thus, our study reveals a novel regulatory mechanism of a key component of the mTOR signaling pathway at the level of gene expression.

## Background

The mechanistic target of rapamycin (mTOR) signaling is involved in the regulation of a broad range of major cellular functions. It integrates various environmental signals such as growth factors, energy or stress levels, and availability of amino acids or oxygen, and in turn, upon the change of those inputs, controls protein and lipid synthesis, energy metabolism, autophagy, lysosome biogenesis, cell survival, and actin cytoskeletal structures [1,2]. Thus it is not surprising that dysfunction of this signaling has been implicated in a variety of pathological conditions including cancer, neurodegeneration, or type 2 diabetes [2,3]. The signal propagation between the pathway components is mainly mediated by phosphorylation events and thus extensive studies have put efforts into deciphering the phosphorylation cascades, constructing pathway maps, and determining the signaling outcomes. One such mTOR pathway branch is insulin signaling; insulin captured by the insulin receptor (InR) at the cell surface triggers the phosphoinositide 3-kinase (PI3K)-Akt phosphorylation cascades and leads to the phosphorylation of the TSC1/2 tumor suppressor complex thereby weakening its GTPase activating protein (GAP) activity towards the small GTPase Rheb. The GTP-bound Rheb then activates the key regulatory kinase TOR complex 1 (TORC1) that phosphorylates the translational repressor 4E-BP (eukaryotic translation initiation factor (eIF) subunit 4E-binding protein) and up-regulates protein synthesis [4]. It is, however, conceivable that the magnitude of the signaling outputs is also affected by the expression levels of the pathway components. The regulatory mechanisms by which the expression levels of these components are determined are still poorly understood.

The Ccr4-Not complex is an enigmatic multi-functional protein complex that is highly conserved throughout the eukaryotic kingdom [5,6]. A role of the protein complex in transcriptional regulation was first discovered in yeast by genetic screens [7,8,9]. A more mechanistic insight was obtained by genetic and biochemical interactions between the complex and subunits of the general transcription factor TFIID that contains the TATA box binding protein (TBP) and the TBP-associated factors (TAFs) [10]. It was subsequently demonstrated that the subunit Not5 recruits TAF1 to promoter DNA [11] suggesting that the Ccr4-Not complex regulates the distribution of TFIID to promoters. However, the Ccr4-Not complex has also been described to exert indirect effects on the control of transcription. One such example is that the complex regulates stability of the Jhd2 demethylase and consequently affects transcription by changing the methylation status of histones at promoters [12]. Interactions between the Ccr4-Not complex and subunits of the SAGA histone acetyltransferase (HAT) complexes were also identified exacerbating mechanistic interpretations [13]. Another functionality was added by the discovery that Ccr4 and Caf1 are deadenylases [14]. Deadenylation is the first step in the exonucleolytic mRNA decay pathway. It was shown that the deadenylase was directed to specific mRNAs through association with RNA binding proteins [15,16,17,18]. In addition, the Ccr4-Not complex was also shown to be recruited to specific mRNAs by microRNAs (miRNAs) [19,20]. Thus, new functions have been constantly added to this protein complex in various eukaryotic organisms: its currently attributed biological roles include mRNA degradation, translational repression, mRNA export from nucleus, DNA damage response, chromatin modification and protein ubiquitination in addition to transcriptional regulation [5]. Despite the broad range of functionalities, only two enzymatic activities have been specified to subunits of the complex so far: a deadenylation activity mediated by the subunits Ccr4 and Caf1 (Twin and Pop2 in *Drosophila*) and an ubiquitination activity intrinsic to Not4 (Cnot4 in *Drosophila*) [14,21,22]. The molecular mechanisms that explain the multi-functionality of this complex have been elusive and its possible target genes are broad [23]. A possible link to mTOR signaling has been suggested by demonstrating that deletion of Ccr4 in a yeast strain increases sensitivity to rapamycin, a drug known to suppress mTOR signaling [24].

Here we show that the Ccr4-Not complex controls the gene expression of 4E-BP, a key mTOR signaling component, at at least two levels, transcription and RNA stability. We have investigated the molecular mechanisms by using reporter expression constructs in which distinct gene regions of the reporter construct are swapped with the counterparts of the endogenous *4E-BP/Thor* gene. We demonstrate that the Ccr4-Not complex regulates both the transcription and mRNA degradation of *4E-BP* gene, suggesting that the balance between transcription and mRNA decay determines the degree of the total effect on expression levels of 4E-BP.

## Results

### Ccr4-Not complex modulates 4E-BP phosphorylation levels

**Fig 1:**
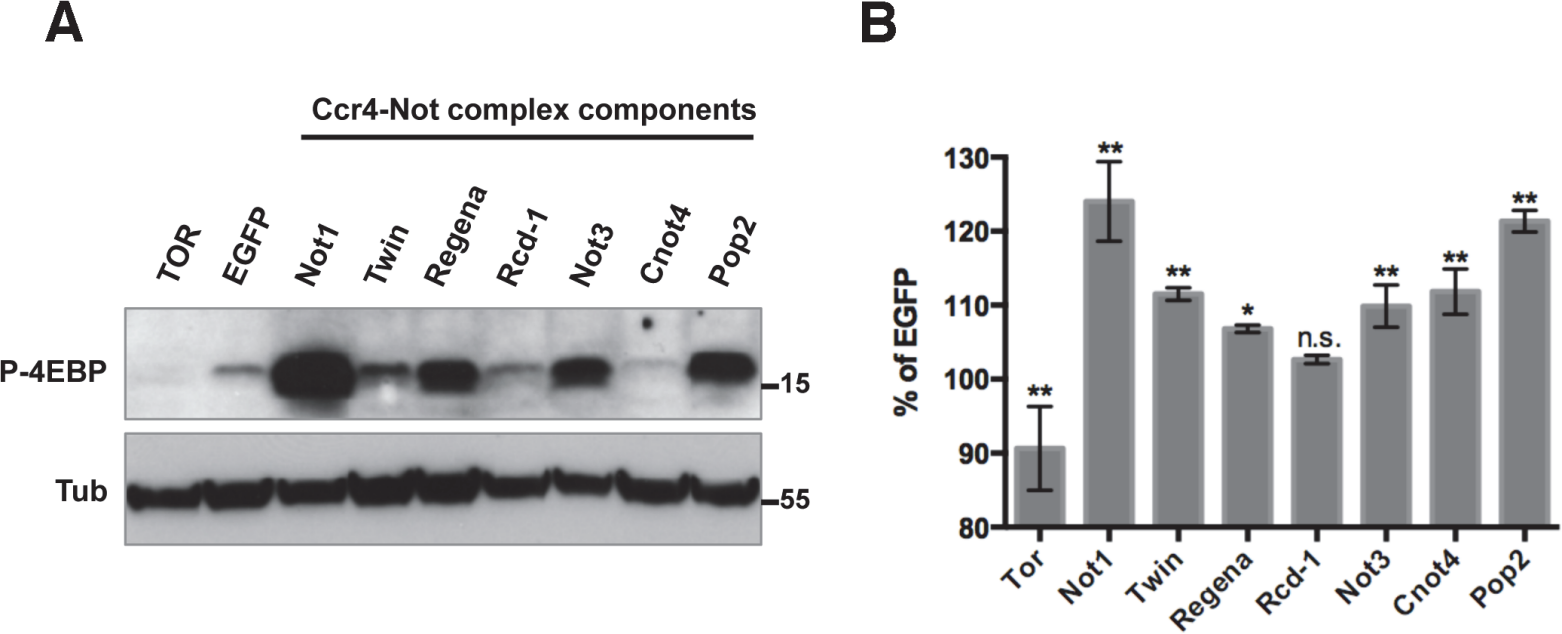
Depletion of Ccr4-Not complex components in Kc cells increases phosphorylation of 4E-BP and cell size upon insulin stimulation. **A**. Increase in insulin-induced 4E-BP phosphorylation by RNAi knockdown of Ccr4-Not complex components. Kc cells were subjected to RNAi treatments against the indicated genes and stimulated with insulin 30 min before cell lysis. The lysates were analyzed by Western blotting using antibodies against anti-phospho 4E-BP and tubulin. **B**. Increased cell size caused by RNAi knockdown of Ccr4-Not complex components. Kc cells were treated as in A. The cell size (area) was measured from three independent experiments and normalized to control (EGFP RNAi). Data are presented as Means ± SEM.

RNAi knockdown of the Ccr4-Not complex subunit Not1 increases the insulin-dependent phosphorylation of the TOR kinase target 4E-BP in *Drosophila* Kc cells (Fig. 1A). This protein complex consists of seven, highly-conserved subunits [6,25] (See Table 1). Similarly, the knockdown of five out of six tested other Ccr4-Not complex components also results in increased 4E-BP phosphorylation (Fig. 1A). We conclude that the Ccr4-Not complex represses 4E-BP phosphorylation upon insulin stimulation in Kc cells. One of the physiological effects of increased 4E-BP phosphorylation is the increase in cell size [4]. As expected, an increase in cell size was observed after knockdown of most Ccr4-Not complex components (Fig. 1B).

**Table 1:** Subunits of *Drosophila* Ccr4-Not complex.

Gene Name/Symbol	Also known as	Annotation Symbol	FlyBase ID	Function Identified *
Not1	CG1884, CG1874	CG34407	FBgn0085436	
Regena	NOT2	CG2161	FBgn0017550	
Not3	l(2)NC136, NOT3/5	CG8426	FBgn0033029	
Cnot4	NOT4, CG5251	CG31716	FBgn0051716	Ubiquitination
Pop2	CAF1	CG5684	FBgn0036239	Deadenylation
Twin	CCR4	CG31137	FBgn0011725	Deadenylation
Rcd-1	CAF40	CG14213	FBgn0031047	

* See references [14,21,22]

### Ccr4-Not complex controls mRNA levels of 4E-BP

**Fig 2:**
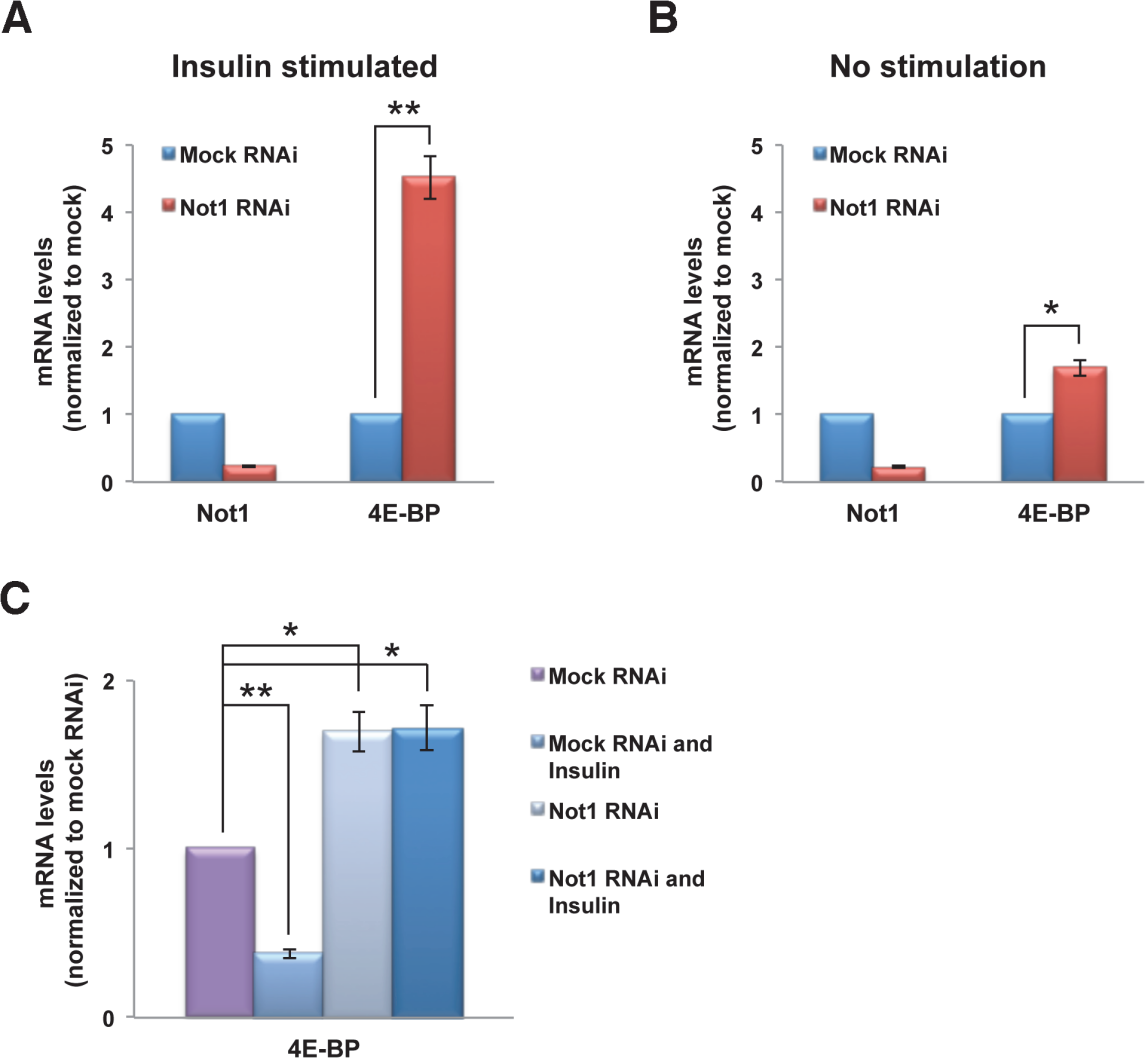
Not1 depletion changes the mRNA level of 4E-BP. Kc cells were treated with Not1 or mock (EGFP) RNAi and mRNA levels of the indicated genes were measured by qPCR and normalized to mock RNAi. Data are represented as Means ± SEM from three independent experiments. **A**. Increased 4E-BP mRNA levels after Not1 knockdown and insulin stimulation. Kc cells were insulin-stimulated 30 min before lysis. **B**. Increased 4E-BP mRNA levels after Not1 knockdown without concomitant stimulation. **C**. Combined effect of insulin stimulation and Not1 RNAi on 4E-BP mRNA levels.

No kinase activity has been identified for the complex. We therefore reasoned that the phosphorylation levels of 4E-BP increased as a mere result of elevated 4E-BP transcript and hence protein levels. This notion is supported by the fact that mRNA degradation has been reported as one of the main functions of the Ccr4-Not complex [26,27,28,29,30]. Indeed, Not1 RNAi treatment increased the 4E-BP mRNA levels 5-fold compared to mock (EGFP) RNAi (Fig. 2A), consistent with the hypothesis that the Ccr4-Not complex degrades 4E-BP mRNA. This effect seems modulated by insulin. In the absence of insulin stimulation, we observe an mRNA increase of 4E-BP of less than two fold (Fig. 2B).

To clarify the underlying mechanism, we separately evaluated the effect of insulin stimulation. Interestingly, insulin stimulation led to a more than 50% decrease in 4E-BP mRNA levels compared to unstimulated cells (Fig. 2C). This reduction is consistent with a previous study in which transcription of 4E-BP was shown to be regulated by the forkhead-related transcription factor FOXO that was inactivated by insulin-induced phosphorylation by Akt [31]. This observation clarifies that the 5-fold difference in 4E-BP mRNA levels in Fig. 2A is caused by the reduction of 4E-BP mRNA by insulin signaling. Not1 reduction, therefore, gives rise to similar levels of 4E-BP mRNA increase irrespective of the presence and absence of insulin stimulation when compared to the same control (mock RNAi with no stimulation) case (Fig. 2C). Hence, it seems that the increase in 4E-BP mRNA caused by Not1 knockdown is not affected by activation of insulin signaling.

### 3’UTR of the 4E-BP gene is critical in Not1-dependent 4E-BP mRNA decay

**Fig 3:**
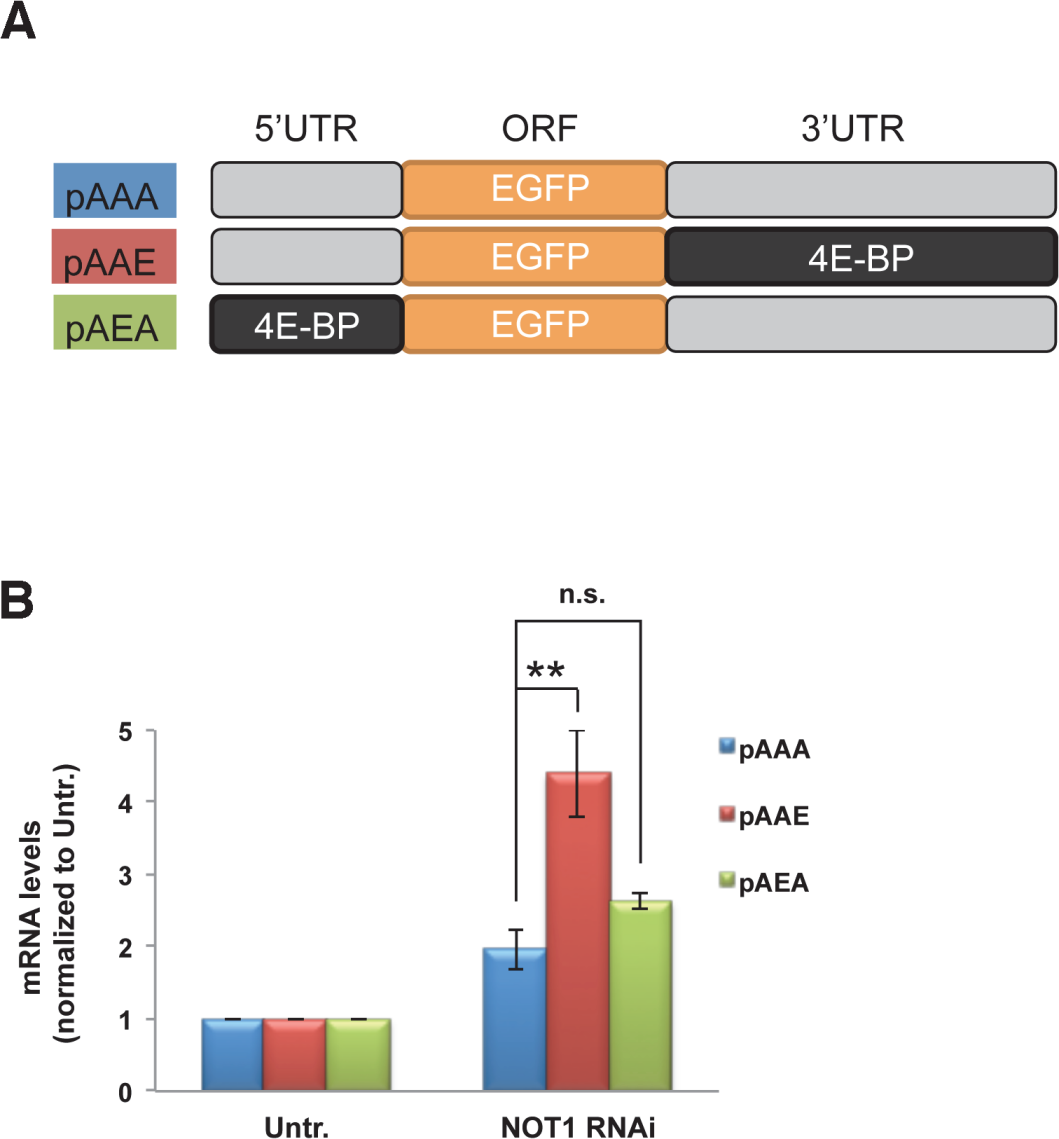
Not1 reduction increases 4E-BP mRNA through the 3’UTR of 4E-BP gene. **A**. Schematic illustration of the reporter constructs in which the 5’UTR or 3’UTR was exchanged with the counterpart of 4E-BP gene. **B**. Changes of the reporter mRNA levels by Not1 depletion. Cells were transfected with the reporter constructs and subjected to Not1 RNAi. After serum starvation overnight and 30 min insulin stimulation, mRNA levels of reporter constructs were measured by qPCR and normalized to untreated (no RNAi/insulin). Data are represented by Means ± SEM from three independent experiments.

To investigate the mechanism by which Not1 controls the mRNA level of 4E-BP, we examined Not1-dependent *4E-BP* gene expression using reporter constructs (See S2 Fig. and S1–S4 Tables). Employing an EGFP-expressing vector as the control reporter expression construct (named pAAA), we created two reporter constructs (pAAE and pAEA) by swapping the 5’UTR and 3’UTR of the control construct with the appropriate counterparts of the *4E-BP* gene, respectively (Fig. 3A). The reporter mRNA levels of the Kc cells depleted with Not1 were normalized to the ones from untreated cells (Fig. 3B). Not1 depletion increased the mRNA levels of the pAAE construct (4E-BP 3’UTR) about twice in comparison to the mRNA levels of the pAAA control construct. However, Not1 depletion did not change mRNA level of the construct carrying the 4E-BP 5’UTR (pAEA). These results indicate that the Ccr4-Not complex reduces the 4E-BP mRNA level by affecting the 3’UTR of the *4E-BP* gene. Consistent with this observation, it has been previously shown that the Ccr4-Not complex is recruited to the 3’UTR of certain mRNAs and exerts a function on poly(A) tail shortening by its deadenylation activity that is essential for mRNA turnover [17,18,32,33,34]. Our results therefore suggest that the Ccr4-Not complex positively regulates the degradation of 4E-BP mRNA.

### Ccr4-Not complex positively regulates the transcription of 4E-BP

**Fig 4:**
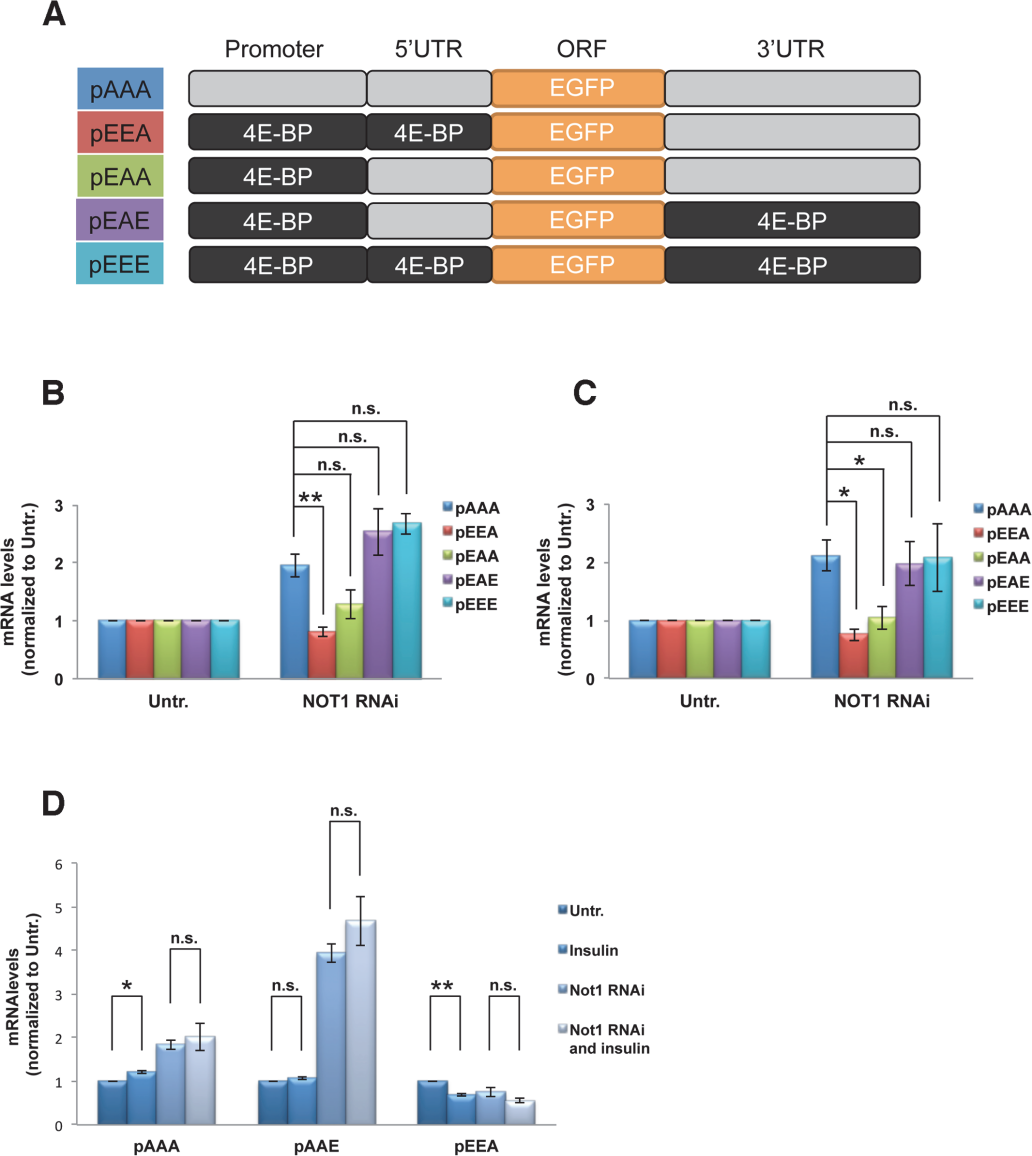
Not1 reduction decreases 4E-BP mRNA through the promoter region of 4E-BP gene. **A**. Schematic of reporter constructs in which the promoter region, 5’UTR and/or 3’UTR were swapped with the counterpart(s) of 4E-BP gene. (see 3B). **B**. Changes of the reporter mRNA levels by Not1 depletion. Cells were subjected to Not1 RNAi and transfection with same amounts of reporter constructs. After serum starvation overnight and 30min insulin stimulation, mRNA levels of reporter constructs were measured by qPCR and normalized to untreated (no RNAi/insulin). Data are represented by Means ± SEM from three independent experiments. (see 3B). **C**. Changes of the reporter mRNA levels by Not1 depletion in the absence of insulin stimulation. The experiment was performed as in B except the absence of insulin addition. **D**. Combined effect of insulin stimulation and Not1 depletion on the reporter mRNA levels. The mRNA levels of the indicated constructs were normalized to untreated (no RNAi/insulin).

To examine the possibility whether the Ccr4-Not complex is also involved in the transcription of *4E-BP*, we created another series of reporter constructs containing various combinations of *4E-BP* gene regulatory regions including its promoter region (Fig. 4A). The presence of the promoter region of the *4E-BP* gene (pEEA and pEAA) caused a reduction in the mRNA levels upon Not1 depletion irrespectively of the 5’UTR suggesting that the Ccr4-Not complex positively regulates the transcription of 4E-BP (Fig. 4B). The reporter constructs pEAE and pEEE, which contain both the 4E-BP promoter region and the 3’ UTR, showed mRNA levels in between those of the 4E-BP promoter and the 4E-BP 3’UTR alone, indicating that the Ccr4-Not complex controls both the transcription and decay of 4E-BP mRNA. The same experiments in the absence of insulin stimulation resulted in very similar levels of reporter mRNAs (Fig. 4C) suggesting that activation of insulin signaling is not necessary for the transcriptional reduction of 4E-BP mRNA. To more carefully examine effects of Not1 depletion and insulin addition separately, the changes of the (representative) reporter mRNA levels induced by Not1 depletion and/or insulin stimulation were compared by normalizing to Untr. (no treatment) (Fig. 4D). A moderate increase of control pAAA mRNA by Not1 depletion is observed but it is not sure whether this increase is due to cellular stress from long-term double-stranded RNA (dsRNA) incubation or due to real Not1 RNAi effect on the control construct. However, the much larger increase in pAAE (4E-BP 3’UTR) mRNA and the decrease in pEEA (4E-BP promoter + 5’UTR) mRNA confirmed a 4E-BP-specific mRNA regulation (mRNA decay and transcription) by the complex. The effect of insulin addition was undetectable, which further supports the notion that insulin signaling does not affect the functions of the complex. The exception is that insulin addition reduced pEEA mRNA level that reflects the insulin-induced FOXO-mediated transcriptional reduction of 4E-BP. In summary, Ccr4-Not complex affects both the generation and clearance of 4E-BP mRNA and consequently causes a reducing effect on mRNA as a total.

### Translational regulation of 4E-BP by Ccr4-Not complex is not detected

**Fig 5:**
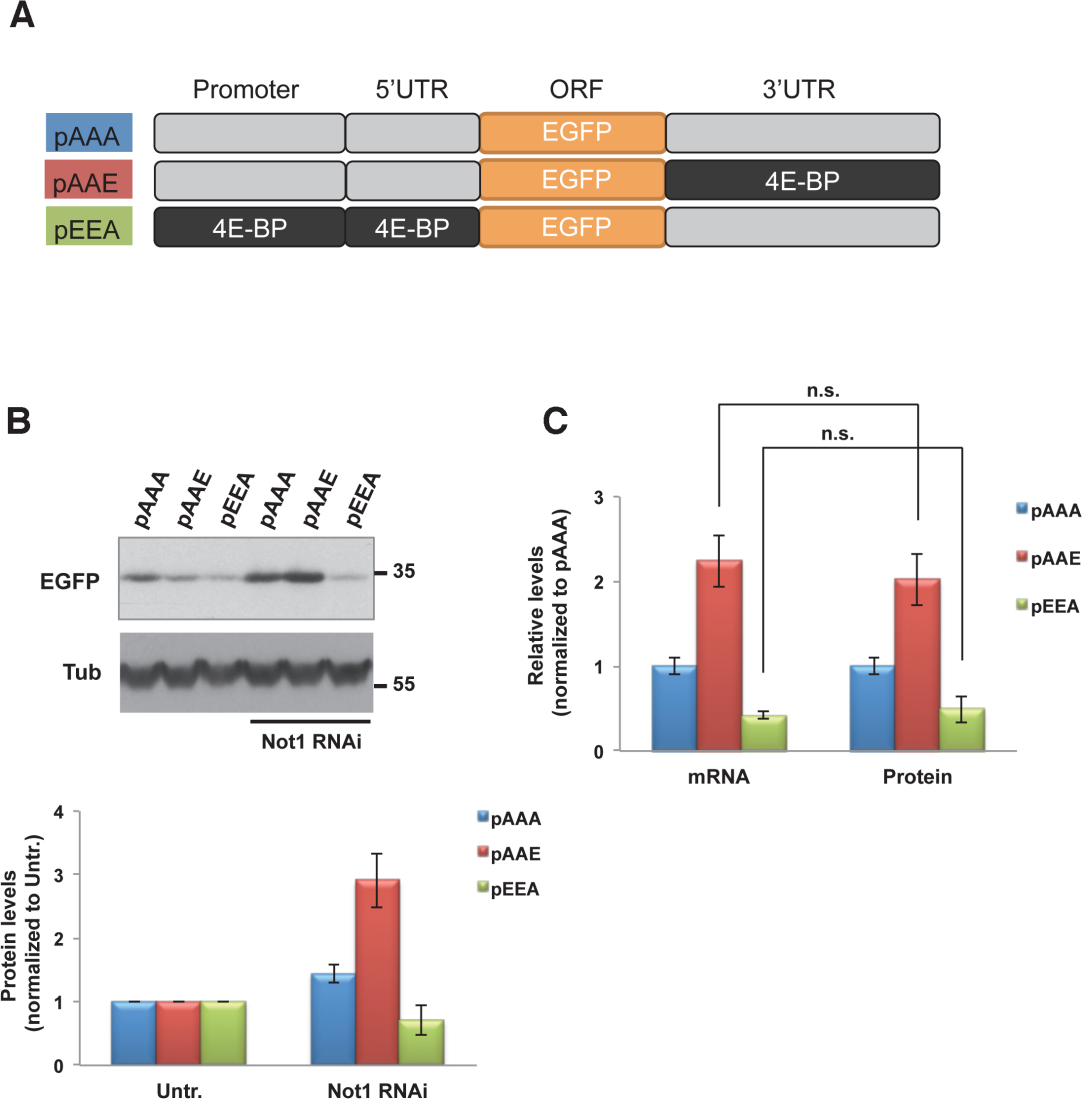
Regulation of 4E-BP translation by Ccr4-Not complex is not detected. **A**. Schematic of reporter constructs that were used for protein level assessment. **B**. Effect of Not1 reduction on the protein levels of the reporter constructs. Cells were under Not1 RNAi and transfection with the indicated constructs. After serum starvation overnight and insulin stimulation for 30 min, cells were lysed and subjected to Western blotting analysis using antibodies against GFP and tubulin. The GFP protein levels (normalized to tubulin) were densitometrically quantified (Image J) from two independent experiments and normalized to untreated (no RNAi/insulin). Means ± SEM are shown. A representative blot is shown. **C**. Relative mRNA and protein levels of the reporter constructs normalized to pAAA.

Translational inhibition by Ccr4-Not complex has been implicated. Poly(A) tails are considered to have a role in the initiation of translation by recruiting poly(A) binding proteins that interact with eukaryotic initiation factor 4G bound to 5’ cap of the mRNA [35,36,37]. Poly(A) shortening by Ccr4-Not complex can be connected to translational repression. In addition, Ccr4-Not complex has been shown to interact with decapping factors [38,39,40]. To investigate the possible effects of Not1 on the translation of the 4E-BP gene, we determined the protein levels of the reporter constructs (Fig. 5A). Importantly, since all reporter constructs produce EGFP proteins, any effects of the Ccr4-Not1 complex on the 4E-BP protein (e.g. degradation via ubiquitination) are eliminated. Thus, significant differences between the EGFP protein levels and the EGFP mRNA levels of each reporter construct can be interpreted as Not1-specific alterations in the translation of the 4E-BP mRNA. Cell lysates were analyzed by Western blotting using a GFP antibody (Fig. 5B). Not1 knockdown increased the EGFP protein levels of the construct carrying the 4E-BP 3’UTR (pAAE), but decreased the EGFP protein levels of the construct containing the 4E-BP promoter region and 5’UTR (pEEA), which is reminiscent of the corresponding mRNA levels. Indeed, comparative analysis between the appropriate EGFP mRNA and protein levels showed that the relative differences between the constructs are mostly identical (Fig. 5C). The same experiments were performed in the absence of insulin (S1A and B Fig.), which did not make differences between the protein and mRNA levels of the reporter constructs. Thus, our study did not identify 4E-BP-specific translation control by Ccr4-Not complex.

## Discussion

**Fig 6:**
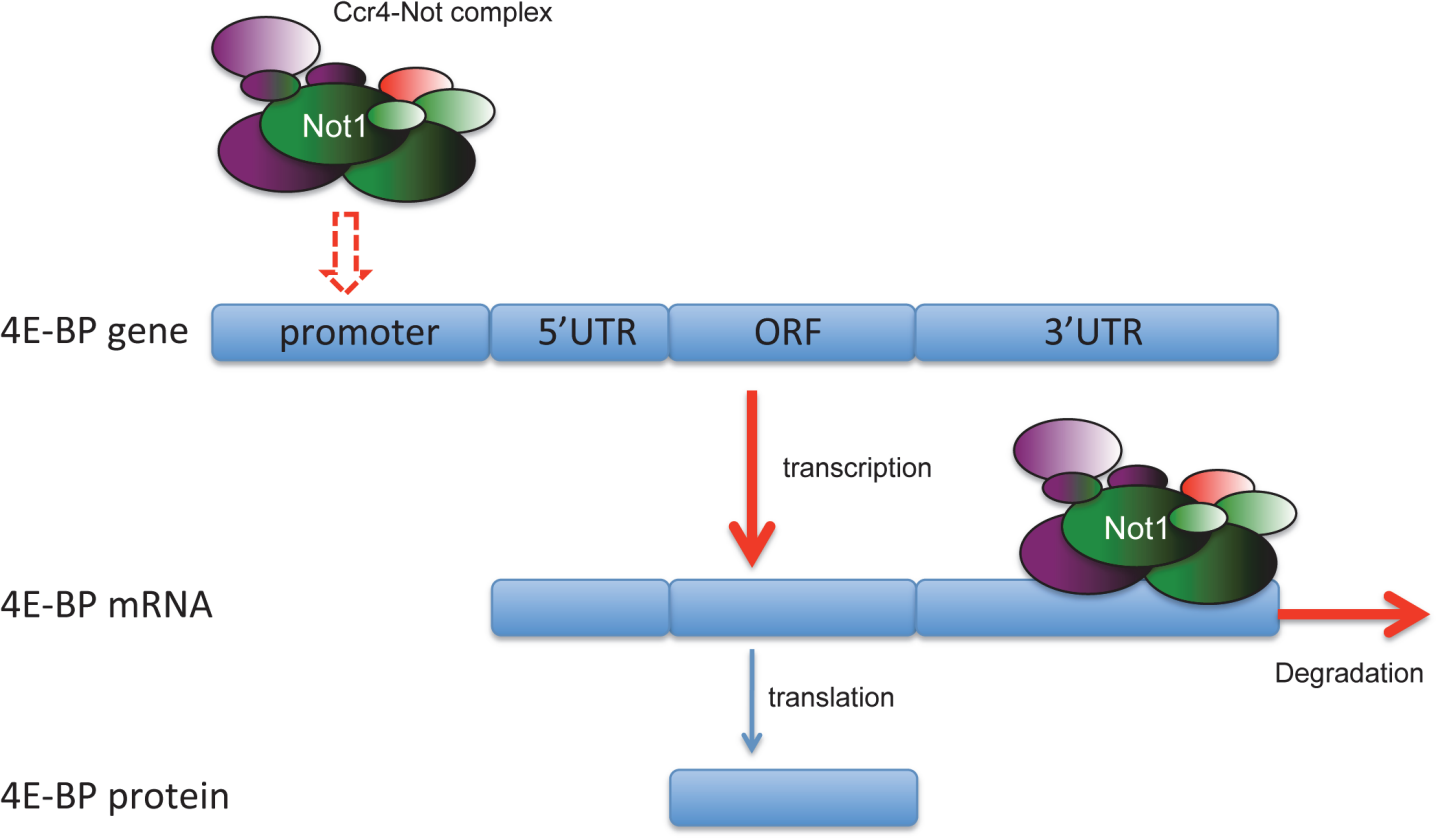
Multi-functional regulation of 4E-BP gene expression by Ccr4-Not Complex. This figure summarizes the current working hypothesis of how the Ccr4-Not complex regulates the expression of 4E-BP. The balance between transcriptional regulation and mRNA decay exerted by the Ccr4-Not complex determines—at least in part—the total protein levels of 4E-BP. Whether the transcriptional control by the complex is mediated by direct binding to the promoter or by indirectly controlling other factors such as transcription factors is not yet clear.

This study describes a novel layer in the regulation of mTOR signaling. We show that the Ccr4-Not complex is involved in controlling the mRNA level of 4E-BP, a critical component of the mTOR signaling cascade. The close examination of the mechanism utilizing reporter mRNAs revealed that the Ccr4-Not complex adjusts the mRNA level of 4E-BP by controlling the balance between its generation and clearance (Fig. 6). However, we have not yet understood whether the complex regulates transcription of 4E-BP in a direct or indirect fashion. Previous studies identified interactions between subunits of the complex and subunits of the transcription factor TFIID containing the TATA-box binding protein TAP and the SAGA histone acetyltransferase complex, suggesting a complex mechanism of transcriptional regulation by the complex that involves multiple factors. However, there is another possibility that the complex indirectly affects the 4E-BP transcription through modulating the functions of transcription factors including FOXO. Thus, the mechanism of 4E-BP transcription by the complex is not yet fully understood. It is of interest to investigate if the Ccr4-Not complex regulates the transcription and degradation of other mTOR signaling pathway components as well. We also investigated possible effect of the Ccr4-Not complex on the translation of 4E-BP, but our assay did not detect any 4E-BP-specific translational regulation by the complex. We were not able to assess effects of Ccr4-Not complex on the protein level of 4E-BP because of the unavailability of good antibodies against *Drosophila* 4E-BP protein. Regulation of the protein stability might be possible through the ubiquitination activity of the Ccr4-Not complex. Considering the diverse functions of the Ccr4-Not complex, it is still possible that Ccr4-Not complex gives other levels of regulation to 4E-BP in addition to mRNA level control.

We have shown that Ccr4-Not complex controls the gene expression profile of the major component in mTOR signaling pathway. We do not know what the function of the differential regulation at the expression and RNA stability level is. It is likely, however, that this novel level of regulation contributes to the complexity of environmental responses that mTOR signaling integrates. Ccr4-Not complex also regulates many other genes. Therefore, we have to be cautious in arguing that phenotypic changes caused by Ccr4-Not complex modulation are due to the change of mTOR signaling. We showed that reduction in Ccr4-Not complex components resulted in an increase in cell size. However, whether the cell size change is mediated by amplified mTOR signaling or through any other changes caused by Ccr4-Not complex depletion is still unclear. In fact, the extent of increase in the 4E-BP phosphorylation did not perfectly correlate with the increased levels of cell size (Fig. 1). For instance, Cnot4 knockdown did not change the 4E-BP phosphorylation but increased cell size slightly, suggesting another effect of Ccr4-Not complex on a distinct mechanism that controls cell size. Further studies are necessary to determine if any other mTOR signaling components are regulated by Ccr4-Not complex at the level of gene expression and how the regulation affects the diverse outputs of mTOR signaling and the resulting phonotypic outcomes.

## Materials and Methods

### Reagents and cell line

Antibodies against phospho 4E-BP1 (Thr37/46), GFP and Tubulin were purchased from Cell Signaling Technology, Life Technologies and Sigma, respectively. Insulin solution (human) was purchased from Sigma. *Drosophila* Kc cells were maintained in Schneider’s Drosophila medium supplemented with 10% fetal bovine serum (FBS) plus penicillin / streptomycin (Life Technologies).

### Plasmids

All EGFP expression reporter constructs created in this study were derived from the *Drosophila* Gateway (Life Technologies) Vector *pAGW*, which expresses EGFP under the control of the *Actin5C* promoter, the *Actin5C* 5’UTR and the *SV40* 3’UTR. Using conventional cloning methods, these regions were swapped with the appropriate counterparts of the *4E-BP* gene resulting in EGFP expression reporter constructs carrying various combinations of regulatory elements from either *4E-BP* and/or *Actin5C/SV40*. To simplify the nomenclature, these constructs were abbreviated with a three-digit code containing either “E” (*4* *E-* *BP*) or “A” (*A* *ctin5C/SV40*) at each position (S1 Table). For example, the abbreviation “EAE” refers to the construct which expresses EGFP under the control of the *4* *E-* *BP* promoter (1^st^ digit), the *Actin5C/SV40* 5’UTR (2^nd^ digit) and the *4* *E-* *BP* 3’UTR (3^rd^ digit). Importantly, “Assembly PCR reactions” (i.e. PCR reactions that use overlapping PCR fragments as template) have been exploited to ensure identical junctions between the different regulatory regions and the EGFP ORF thereby eliminating position effect variations. A detailed description of the cloning strategies as well as of all primers and PCR reactions is provided in the Supporting Information (S2 Fig., S2–S4 Tables).

### Double-strand RNA preparation

Genomic DNA of fly was first prepared as previously described [41]. Briefly, one fly was homogenized in 50ul of squishing solution (200ug/ml of Proteinase K in10mM Tris-HCl pH8.2). After incubation at 37°C for 30 minutes and subsequent heat inactivation at 95°C for 2–5 minutes, the supernatant was collected following a quick spindown. Next, the target DNA sequence was amplified by standard PCR with Phusion polymerase using 1–2ul of the purified genomic DNA (or otherwise plasmids that contain the cDNA sequences of target genes in case of Tor and EGFP). The following primers are used for Not1 (5’-CGCTAATACGACTCACTATAGGGAGAACATAGCACACAGCGCCCAC-3’ and 5’-CGCTAATACGACTCACTATAGGGAGATGCACAAAGTCATGGTCCCA-3’), Tor (5’-CGCTAATACGACTCACTATAGGGAGAATGTCGACGACGTCGGTGG-3’ and 5’-CGCTAATACGACTCACTATAGGGAGAGGCAAGGCGATAGCCAGC-3’), Twin (5’-CGCTAATACGACTCACTATAGGGAGAGCACTATGCAGCGGACATTA-3’ and 5’-CGCTAATACGACTCACTATAGGGAGACGACGTACTTCCTCTCCAG-3’), Rcd-1 (5’-CGCTAATACGACTCACTATAGGGAGAGCTGCTCAAGAACCTGGAAC-3’ and 5’-CGCTAATACGACTCACTATAGGGAGATCATCGATGGTTGGACGTTA-3’), Cnot4 (5’-CGCTAATACGACTCACTATAGGGAGACTAGCAATAGAACGAGGGCG-3’ and 5’-CGCTAATACGACTCACTATAGGGAGACACTTTCTTGCTGGCTTTCC-3’), Regena (5’-CGCTAATACGACTCACTATAGGGAGAAATGGTCTCGGTGCCGTTG-3’ and 5’-CGCTAATACGACTCACTATAGGGAGACGAAGTTTGCACGCCGCTC-3’), Not3 (5’-CGCTAATACGACTCACTATAGGGAGATAGGCTGCTTGACAATGACG-3’ and 5’-CGCTAATACGACTCACTATAGGGAGATGCTCTTGTTGCGTTTATCG-3’), Pop2 (5’-CGCTAATACGACTCACTATAGGGAGATCCAGCACTTGAATCGAAGAG-3’ and 5’-CGCTAATACGACTCACTATAGGGAGAGACCGTGTAGGTTTCGGC-3’), EGFP (5’-CGCTAATACGACTCACTATAGGGAGATCACCGGGGTGGTGCCCATCCTGG-3’ and 5’-CGCTAATACGACTCACTATAGGGAGATGCCGAGAGTGATCCCGGCGGCGG-3’). Note the above primers contain T7 promoter sequence at the 5’ end. After purification of the PCR product using NucleoSpin Gel and PCR Clean-up (Macherey-Nagel), double-strand RNA was synthesized by in vitro transcription using Ambion Megascript kit following manufacturer’s instruction (Ambion).

### RNAi and transfection

Three millions of cells per well were seeded in 1 ml of serum-free Schneider’s media in 6-well plates and mixed with 15 μg of double-stranded RNAs against the gene of interest. After 30 minutes of incubation at 25°C, 2 ml of Schneider’s media containing 15% FBS plus penicillin/streptomycin was added. After four days of incubation at 25°C, cell media was replaced with serum-free Schneider’s media. The next day, cells were harvested, washed with cold PBS, and kept at −80°C until use. In case of no serum starvation, cells were incubated for five days prior to harvest. When cells were stimulated with insulin, insulin solution (Sigma) was added to cell media 30 minutes prior to harvesting cells at 1:1000 dilution (10 μg/ml) following the manufacturer’s instruction.

In case of reporter plasmid transfection, Effectene reagent (Qiagen) was used. Two days after RNAi treatment, 1ml of cell media was first removed to reduce the media to 2ml. Transfection of cells with a reporter construct was then performed following the Effectene manufacture’s instruction, which was followed by a direct addition (without help of Effectene) of 5 μg double-strand RNA into cell media. After two days of incubation at 25°C, cell media was replaced with serum-free Schneider’s media, followed by insulin treatment and harvest the next day.

### Real-time PCR experiment

Total RNA was extracted from cells using the RNeasy mini kit (Qiagen). First-strand cDNA was synthesized with Oligo-dT (18 bases) primers using SuperScript III reverse transcriptase (Life Technologies). The PCR reaction was prepared using LightCycler 480 SYBR Green I Master (Roche Applied Science) in combination with target-specific primers. PCR was quantified using Pfaffl method and normalized to mRNA levels of internal controls (glycerol-3-phosphate dehydrogenase (Gpdh) and ribosomal protein RpL23). The primers used to amplify target mRNAs are as follow; Not1 (5’-GCACGTGAACAAGCGAAAT-3’ and 5’-CCAGGCCGTGCTCTTTAAC-3’), 4E-BP (5’-CCAGATGCCCGAGGTGTA-3’ and 5’-AGCCCGCTCGTAGATAAGTTT-3’), Gpdh (5’-AGTACCTGAAAGGACACAAGCTG-3’ and 5’-CCACGAAGATCAGGATGTCA-3’) and RpL23 (5’-TCGTAACGCTCAGCAACG-3’ and 5’-GCAGACCTCTTGGCTTGC-3’).

### Cell size measurement

For size measurements, the cells were washed and resuspended in ice-cold PBS. The forward and sideward scatter of at least 40.000 cells has been measured with a FACSCalibur cell analyzer (BD Biosciences) using predetermined settings suitable for *Drosophila* Kc cells. FlowJo software was used to convert the forward scatter distribution into cell size values (arbitrary units), which were subsequently normalized to the size of EGFP-RNAi treated Kc cells (which was set to 100%). At least three independent experiments have been conducted and averaged for each analysis.

### Statistical analysis

All the statistical significances were tested using Student’s t-test (two-tailed). P-value levels were represented in the figures using the following symbols: *P<0.05, **P<0.01. n.s. indicates no significance.

## Supporting Information

S1 Fig Regulation of 4E-BP translation by Ccr4-Not complex without insulin stimulation. **A**. Effect of Not1 reduction on the protein levels of the reporter constructs. Cells were treated with Not1 RNAi and transfected with the indicated constructs. After serum starvation overnight (but without insulin stimulation), cells were lysed and subjected to Western blotting analysis using antibodies against GFP and tubulin. The GFP protein levels (normalized to tubulin) were densitometrically quantified (Image J) from two independent experiments and normalized to untreated (no RNAi/insulin). Means ± SEM are shown. A representative blot is shown. **B**. Relative mRNA and protein levels of the reporter constructs normalized to pAAA.
(PDF)

S2 Fig Cloning strategies. This figure schematically illustrates the creation of the 7 different EGFP expression reporter constructs used in this study. For each construct, a certain PCR fragment (or Assembly PCR fragment; see S2 and S3 Tables) was used to replace a region in the parental vector, which also eliminated the large gateway cassette. The *dashed lines* indicate the used restriction enzymes. Dimensions are not in scale.
(PDF)

S1 Table EGFP expression reporter constructs. (PDF)

S2 Table PCRs. A detailed description of the PCR reactions in cloning the reporter constructs.
(PDF)

S3 Table Hybrid PCRs. A detailed description of the hybrid PCR reactions in cloning the reporter constructs.
(PDF)

S4 Table Primers used for cloning. (PDF)
