# Supplementary figures and images for: Multi-Functional Regulation of 4E-BP Gene Expression by the Ccr4-Not Complex
Source: PLoS One. 2015 Mar 20;10(3):e0113902. doi: 10.1371/journal.pone.0113902 (PMC4368434; doi:10.1371/journal.pone.0113902)

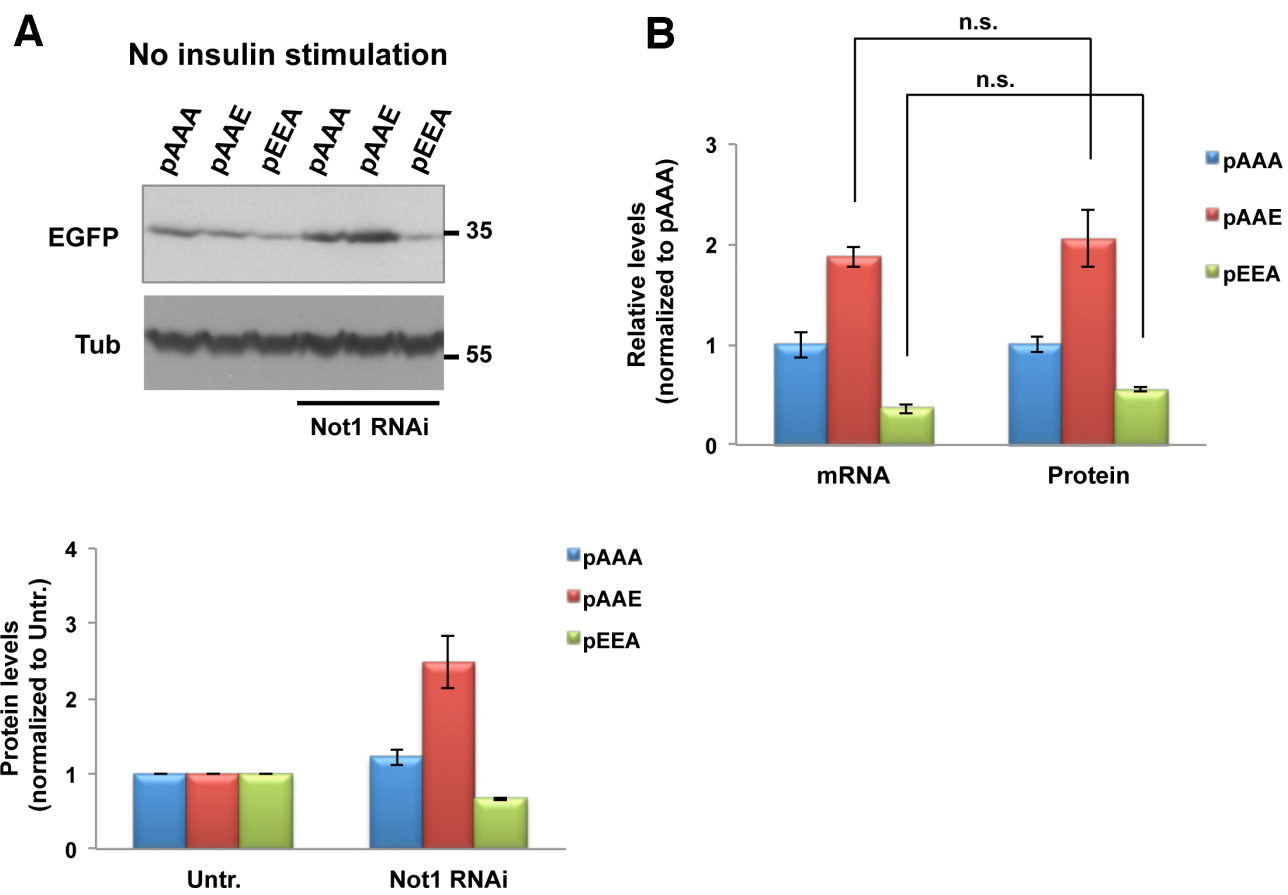

Supplement: S1 Fig — A. Effect of Not1 reduction on the protein levels of the reporter constructs. Cells were treated with Not1 RNAi and transfected with the indicated constructs. After serum starvation overnight (but without insulin stimulation), cells were lysed and subjected to Western blotting analysis using antibodies against GFP and tubulin. The GFP protein levels (normalized to tubulin) were densitometrically quantified (Image J) from two independent experiments and normalized to untreated (no RNAi/insulin). Means ± SEM are shown. A representative blot is shown. B. Relative mRNA and protein levels of the reporter constructs normalized to pAAA. (PDF) [file pone.0113902.s001.pdf]

Supplementary Figure 2

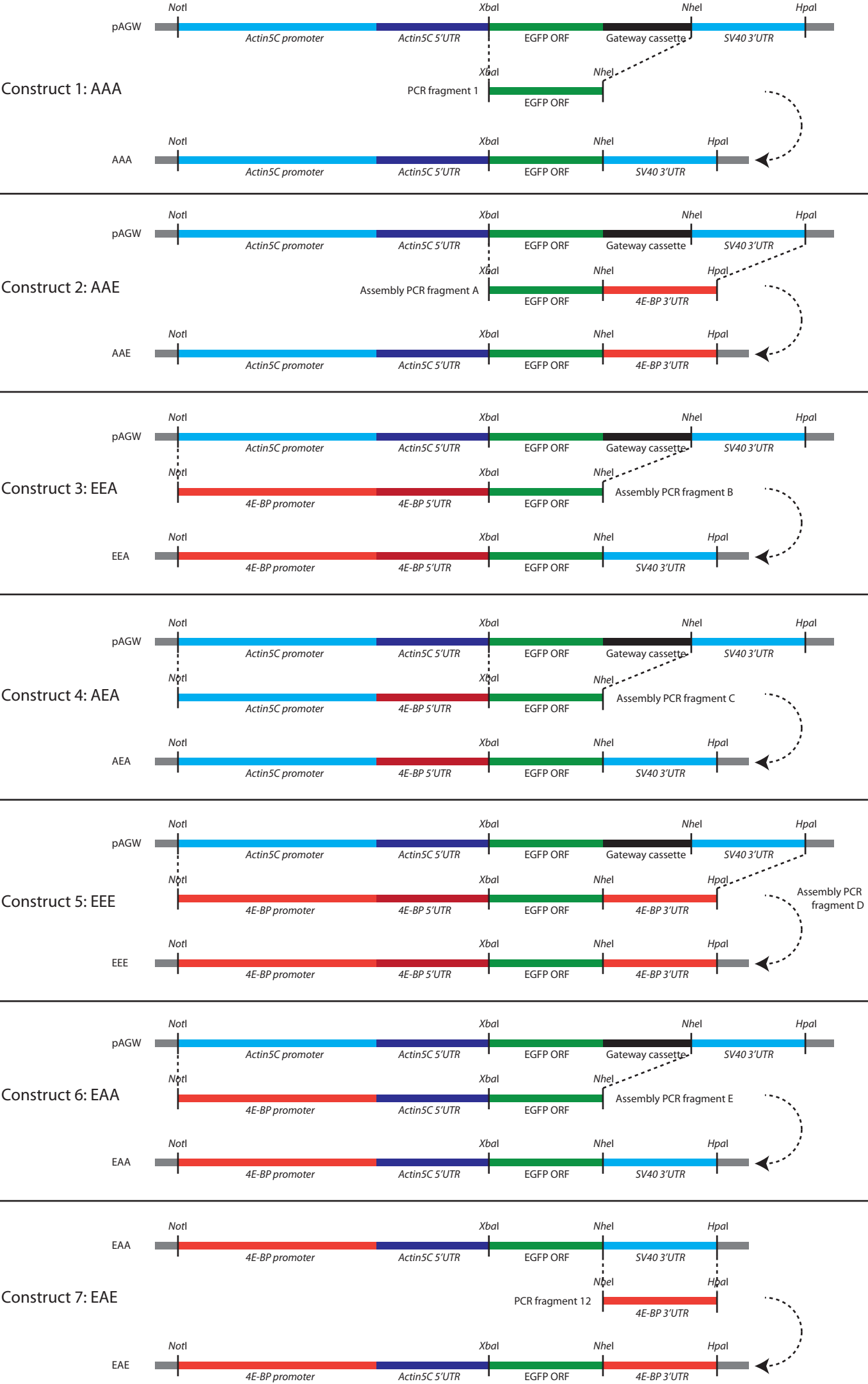

Supplement: S2 Fig — This figure schematically illustrates the creation of the 7 different EGFP expression reporter constructs used in this study. For each construct, a certain PCR fragment (or Assembly PCR fragment; see S2 and S3 Tables) was used to replace a region in the parental vector, which also eliminated the large gateway cassette. The dashed lines indicate the used restriction enzymes. Dimensions are not in scale. (PDF) [file pone.0113902.s002.pdf]
