# Supplementary material for: Multi-Functional Regulation of 4E-BP Gene Expression by the Ccr4-Not Complex
Source: PLoS One. 2015 Mar 20;10(3):e0113902. doi: 10.1371/journal.pone.0113902 (PMC4368434; doi:10.1371/journal.pone.0113902)
Supplement: S1 Table — (PDF) [file pone.0113902.s003.pdf]

Supplementary Table 1

Supplementary table 1: EGFP expression reporter constructs

Abbreviation	Promoter	5'UTR	3'UTR
AAA	Actin5C	Actin5C	SV40
EAA	4E-BP	Actin5C	SV40
AEA	Actin5C	4E-BP	SV40
AAE	Actin5C	Actin5C	4E-BP
EEA	4E-BP	4E-BP	SV40
EAE	4E-BP	Actin5C	4E-BP
EEE	4E-BP	4E-BP	4E-BP
