# Supplementary material for: Multi-Functional Regulation of 4E-BP Gene Expression by the Ccr4-Not Complex
Source: PLoS One. 2015 Mar 20;10(3):e0113902. doi: 10.1371/journal.pone.0113902 (PMC4368434; doi:10.1371/journal.pone.0113902)
Supplement: S2 Table — A detailed description of the PCR reactions in cloning the reporter constructs. (PDF) [file pone.0113902.s004.pdf]

Supplementary Table 2

Supplementary Table 2: PCRs

PCR fragment	Amplification of	Template	Primer
1	EGFP	AAE	RS366 / RS373
2	EGFP	pAGW	RS366 / RS367
3	3'UTR-4EBP	genomic DNA	RS368 / RS369
4	5'UTR-4EBP	genomic DNA	RS370 / RS371
5	EGFP	pAGW	RS372 / RS373
6	Actin5C promoter	pAGW	RS428 / RS429
7	5'UTR-4EBP	EEA	RS430 / RS431
8	5'UTR-4EBP-EGFP	EEA	RS370 / RS425
9	EGFP-3'UTR-4EBP	AAE	RS426 / RS427
10	4EBP promoter	EEA	RS370 / RS432
11	5'UTR-Actin5C-EGFP	pAGW	RS433 / RS431
12	3'UTR-4EBP	AAE	RS440 / RS427
