# Supplementary material for: Multi-Functional Regulation of 4E-BP Gene Expression by the Ccr4-Not Complex
Source: PLoS One. 2015 Mar 20;10(3):e0113902. doi: 10.1371/journal.pone.0113902 (PMC4368434; doi:10.1371/journal.pone.0113902)
Supplement: S3 Table — A detailed description of the hybrid PCR reactions in cloning the reporter constructs. (PDF) [file pone.0113902.s005.pdf]

Supplementary Table 3

Supplementary Table 3: Hybrid PCRs

Hybrid PCR fragment	Amplification of	Template	Primer
**A**	**EGFP-3'UTR-4EBP**	**PCR fragments 2 + 3**	**RS366 / RS369**
**B**	**5'UTR-4EBP-EGFP**	**PCR fragments 4 + 5**	**RS370 / RS373**
**C**	**Actin5CPro-5'UTR-4EBP-EGFP**	**PCR fragments 6 + 7**	**RS428 / RS431**
**D**	**5'UTR-4EBP-EGFP-3'UTR-4EBP**	**PCR fragments 8 + 9**	**RS370 / RS427**
**E**	**4EBPPro-5'UTR-Actin5C-EGFP**	**PCR fragments 10 + 11**	**RS370 / RS431**
