# Supplementary material for: Multi-Functional Regulation of 4E-BP Gene Expression by the Ccr4-Not Complex
Source: PLoS One. 2015 Mar 20;10(3):e0113902. doi: 10.1371/journal.pone.0113902 (PMC4368434; doi:10.1371/journal.pone.0113902)
Supplement: S4 Table — (PDF) [file pone.0113902.s006.pdf]

Supplementary Table 4

Supplementary Table 4: Primers used for cloning

Name	Sequence (5’ > 3’)
**RS366**	**CATCAGCCAGCAGTCGTCTA**
**RS367**	**CATGGCGGTGTACACGCCACACCCCCTACTTGTACAGCTCGTCCATG**
**RS368**	**TCTCGGCATGGACGAGCTGTACAAGTAGGGGGTGTGGCGTGTACAC**
**RS369**	**CTTGTCGTTAACCACATCCGCACCTACGTCTA**
**RS370**	**ACGAGCCACGCGGCCGCCGAGTAACCACAGGCCAGAT**
**RS371**	**ACAGCTCCTCGCCCTTGCTCACCATCTTAGCTGATTGATTGGATTG**
**RS372**	**AAACCAATCCAATCAATCAGCTAAGATGGTGAGCAAGGGCGAGG**
**RS373**	**CTTGTGCTAGCTTACTTGTACAGCTCGTCCATGC**
**RS425**	**CTACTTGTACAGCTCGTCCATG**
**RS426**	**ATGGTGAGCAAGGGCGAGG**
**RS427**	**CTTGTCGTTAACTTTGTCCTATGCGAAACATCG**
**RS428**	**TCATTAATGCAGGCAACTCG**
**RS429**	**GTTGGCTGTTCGCTAGGCGATTGCTAAAACTGGTCCCGCAGCC**
**RS430**	**TAAAACGGGCTGCGGGACCAGTTTTAGCAATCGCCTAGCGAACAG**
**RS431**	**GATCCTGCTAGCTTACTTGTACAGCTCGTCCAT**
**RS432**	**CAAGAACTCAAACGGTAGTGATATGGGCGGTATTACGAAGTGTGG**
**RS433**	**TAGAGCCACACTTCGTAATACCGCCCATATCACTACCGTTTGAGTTC**
**RS440**	**CTGTACGCTAGCGGGGTGTGGCGTGTACAC**
